# Impact of various prognostic factors on survival in glioblastoma: tertiary care institutional experience

**DOI:** 10.3332/ecancer.2022.1386

**Published:** 2022-05-12

**Authors:** Swapna Jilla, Archana Prathipati, Bala Venkata Subramanian, Pranabandhu Das, Deepthi Valiyaveettil

**Affiliations:** 1 Department of Radiation Oncology, Mallareddy Cancer Hospital and Research Institute, Suraram X Road, Jeedimetla, Hyderabad, 500055, India; 2 Department of Radiation Oncology, SLG Hospital, Nizampet, Hyderabad, 500090, India; 3 Department of Radiation Oncology, Sri Venkateshwara Institute of Medical Sciences, Tirupathi, 517501, India

**Keywords:** glioblastoma, prognostic factors, radiation, temozolomide

## Abstract

**Introduction:**

Glioblastoma is the most common malignant brain tumour in adults. Among all gliomas, it is the most aggressive type, with a high fatality rate, and according to the WHO classification, it is a grade IV tumour. As this tumour is well-known for its poor survival, an understanding of clinical and treatment-related prognostic factors can help in tailored treatment. The aim of this study was to know the impact of prognostic factors on survival in these cases.

**Materials and methods:**

All glioblastoma patients treated in our hospital during 2010–2015 were included in the analysis. Cases were divided into different groups based on prognostic factors. Overall survival (OS) and disease-free survival (DFS) were calculated and compared among the different groups. Statistical analysis was carried out using SPSS software v20.

**Results:**

One-year OS was 36.9% and 2-year OS was 10.8%. One-year DFS was 13.04%. On univariate analysis, age at presentation ≤45 years and adjuvant chemotherapy with six cycles or more temozolomide improved OS and DFS. Multivariate analysis retained the statistically significant positive impact of usage of adjuvant temozolomide chemotherapy of ≥six cycles on OS and DFS. The use of the anti-epileptic drug Levetiracetam had a statistically significant improvement of DFS.

**Conclusion:**

Among various clinical and treatment-related prognostic factors evaluated in our study, younger age at presentation and addition of temozolomide chemotherapy to radiation showed improvement in OS and DFS. The use of the anti-epileptic drug Levetiracetam had an impact on DFS in glioblastoma patients.

## Introduction

Glioblastoma is the most common malignant brain tumour in adults. Among all gliomas, it is the most aggressive type, with a high fatality rate, and according to the WHO classification, it is a grade IV tumour [1].

Common symptoms of presentation are headache, vomiting, drowsiness, seizures and neurological deficits. The standard treatment protocol is maximum safe resection, followed by adjuvant concurrent chemoradiotherapy and adjuvant chemotherapy with six cycles of temozolomide. This standard of treatment led to improved survival of patients compared to historical controls. However, most of the patients developed recurrent disease early in the course of treatment due to multifocal stem cell niches, like progenitor cells, and succumbed to the illness in less than 2 years [2]. Recurrent disease is treated with salvage surgery if possible or re irradiation or chemotherapy with six more cycles of temozolomide once in 4 weeks.

Further advances are progressing towards molecular genetics and prognostic factors at the gene level to assess treatment responses and to improve survival. Genetic testing may not be feasible for all patients, especially in low income countries, hence clinical factors like age at presentation, symptom duration and performance status may be important for prognostication before treatment. Several clinical studies have identified factors affecting clinical outcomes [3]. This helps in tailored treatment approaches.

With this background, we have conducted a retrospective analysis to know the impact of various prognostic factors affecting overall survival (OS) and disease-free survival (DFS) in glioblastoma patients treated at our institute.

## Materials and methods

All histopathologically confirmed cases of glioblastoma treated in our hospital during 2010–2015 were included in the analysis.

Complete history and physical examination was conducted for all patients preoperatively. They were evaluated radiologically with CT and MRI brain to know the extent of the lesion. Prognostic factors were documented for all the patients.

### Treatment history

Maximal safe resection was carried out in all patients.

Adjuvant radiation to the brain was given with a total dose of 60 Gray (Gy) in 30 fractions, at a dose of 2 Gy per fraction, with 5 fractions per week with three-dimensional conformal radiotherapy.

Concurrent chemotherapy was given with oral alkylating agent temozolomide at 75 mg/m^2^ body surface area (BSA) for 5 days a week, half an hour before radiation for 6 weeks.

After completion of radiation, adjuvant chemotherapy was continued with temozolomide at 150 mg/m^2^ BSA once a day for 5 days, once in 4 weeks, for six cycles. Response evaluation was carried out with MRI brain, and further six cycles chemo was continued in cases with residual disease. Patients were kept on follow-up.

OS was calculated from the day of surgery till date of death or last follow-up.

DFS was calculated from the date of surgery to the date of recurrence or death.

For analysis, we considered various prognostic factors and grouped them under various categories (Table 1).

### Data analysis

Survival curves were estimated using the Kaplan–Meier method. The effect of prognostic groups on survival was estimated by univariate and multivariate analyses. 95% confidence intervals for the median survival time of the groups were constructed and log-rank test was employed to determine if there is statistical evidence of differences between the survival curves of the groups. Finally, the Cox proportional hazard model was used in the multivariate analysis. Statistical significance was set at *p*-value < 0.05. Statistical analysis was carried out using SPSS V 20 (IBM corp. In Armonk, NY).

## Results

A total of 70 patients were screened, and patients with incomplete data, like preoperative clinical data, surgery details, defaulters for radiation and death during radiation, were excluded, and the remaining 46 cases were included in the analysis.

The mean and median age of presentation in the whole cohort was 48.5 years (range = 21–76 years) and 51 years, respectively. Males presented at a slightly older age compared to females. The mean and median age of presentation among males was 49 and 52.5 years, respectively, while the mean and median age of presentation among females was 47.5 and 50 years, respectively. Complete excision was carried out in 17 patients. Concurrent chemotherapy with temozolomide was given in 31 patients. In the adjuvant setting, 15 patients completed six cycles of adjuvant temozolomide.

### Survival data

One-year OS was 36.9% and 2-year OS was 10.8%. One-year DFS was 13.04% and only 1 patient was disease free at 2 years. The mean and median OS was 10.5 and 8 months, respectively, and the mean and median DFS was 7.5 and 6.5 months, respectively.

### Survival analysis

**Figure 1.**
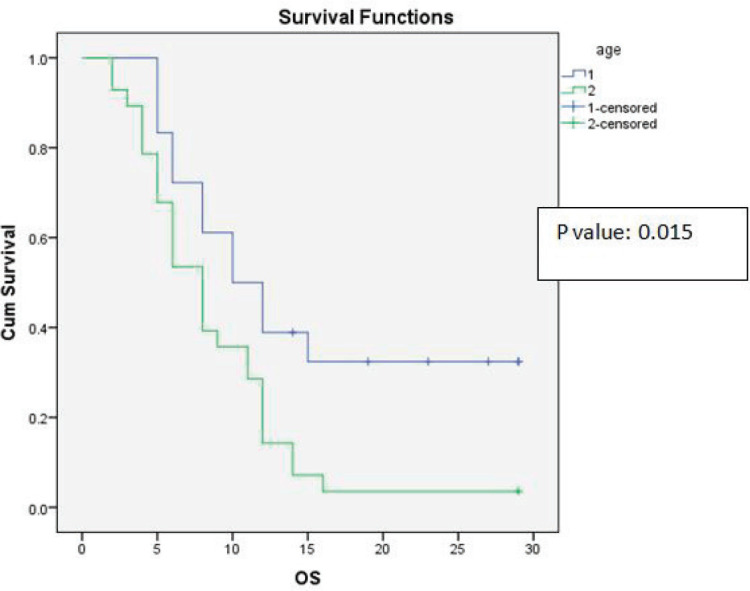
Kaplan-Meier survival curve showing effect of age on OS.

**Figure 2.**
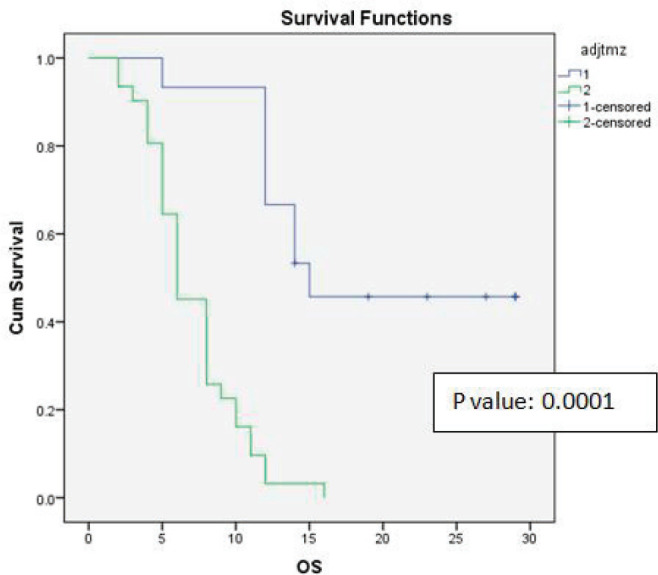
Kaplan-Meier survival curve showing effect of adjuvant temozolomide on OS.

Univariate analysis found a statistically significant correlation between OS at age of presentation and adjuvant chemotherapy with temozolomide. The age at presentation ≤45 years and adjuvant chemotherapy with 6 cycles or more temozolomide showed improved survival (Figures 1 and 2).

**Figure 3.**
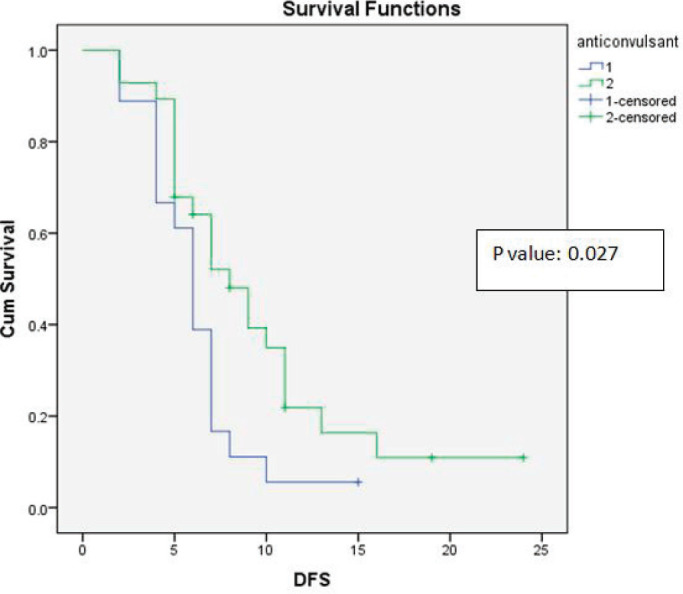
Kaplan-Meier survival curve showing effect of anticonvulsant levetiracetam use on DFS.

Age at presentation, the anti-epileptic drug Leviteracetam and adjuvant chemotherapy with temozolomide showed statistically significant correlations with DFS and OS (Figure 3). The results of the univariate analysis are documented in Table 1.

Multivariate analysis showed a statistically significant impact on OS and DFS in patients using adjuvant temozolomide.

## Discussion

In this study, we analysed the data of newly diagnosed and treated cases of glioblastoma in a tertiary care institute of those who completed treatment as per protocol. Glioblastoma is, according to the WHO, a grade 4 tumour with an incidence of 12%–15% among brain tumours. It presents predominantly in the age group of 45–70 years, with incidences favouring males; it has a male-to-female ratio of 1.5:1 [4].

Our study showed a similar male predominance with a male-to-female ratio of 1.3:1. The mean and median age of presentation of the entire population was 48.5 and 51 years, respectively, which are well within the reported range.

As Lamborn *et al* [5] suggested that the statistically significant impact of younger age improved survival, our study also proved that the significant impact of younger age improved median OS and DFS (*p*-value = 0.015, 0.015) on univariate analysis.

The mean and median OS was 10.5 and 8 months, respectively, and the mean and median DFS was 7.52 and 6.5 months, respectively, in the entire patient population in our study, which is less than the median survival reported in the EORTC-NCIC trial [6] (14.6 and 6.9 months, respectively). The 2- and 5-year survival rates were 27.2% and 9.8%, respectively [6]. But our study data correlates with the Indian data reported by Kumar *et al* [7] in which the median survival is 7.67 months. We reported 1-year OS of 36.9% and 2-year OS of 10.8%, which is little higher than the reported Indian data of 25.63% and 7.23%, respectively [7]. This might be because of the small sample size.

With regard to gender-wise survival, males had a higher mortality and lower survival rate compared with that of females [8], and our study showed a little higher OS trend among females, although not significant (10.75 versus 10.38 months).

In the literature, glioblastoma presenting with seizures remained significantly associated with an increased OS compared with glioblastoma patients without seizures by helping in early diagnosis [9], but in our study, the impact of seizure as a symptom of presentation did not significantly affect OS (mean = 13.21 versus 9.28) or DFS; this may be because, in cases presenting with seizure (14 patients out of 46), only 6 (42.8%) underwent complete excision, with good performance status, and only 7 (50%) received 6 or more cycles of adjuvant chemotherapy, which may have impacted median survival rates.

Location of the tumour may impact prognosis by effecting completeness of surgery, i.e., eloquent locations may not permit radical resections, while non-eloquent and accessible location may permit more radical surgery [10], but our study had not shown any significant impact of cerebral cortex versus subcortical/deep structure locations on OS or DFS. This could be due to the unbalanced sample number in the two groups.

Lesion may be localised on left side or right side of the brain or midline. Some studies showed that left-sided lesions have improved survival [7], while Tait *et al* [11] suggested better survival with right-sided and central tumours. Our analysis had not shown significance of the side of lesion similar to the reported data.

Similarly, lesions localised to frontal lobes express better survival because of the possibility of radical resections [7]. However, our study could not prove this effect on OS or DFS. This could be due to other confounding factors.

It is well known that good performance status will positively impact survival in all age groups, but our study results show a trend of improved survivals and are not statistically significant because of the small sample size [12, 13].

Recent studies have suggested that the usage of the anti-epileptic drug Levetiracetam can inhibit malignant glioma cell proliferation and sensitise glioblastoma cells to temozolomide, thus improving survival [14]. Our study showed a trend towards improved OS and statistically significant improvement in DFS (*p*-value = 0.027).

The importance of surgical completeness was highlighted in many studies. Almenawer *et al* [15] concluded that mean OS was significantly longer with maximum grades of resection. Moreover, patients undergoing complete resection experienced better functional recovery, but the result of our study does not support this evidence and this could be due to many factors, like small sample size, few patients with complete resection (17 versus 29) and poor prognostic factor combinations, neutralising the positive effect of completeness of the surgery.

Delay at the start of radiation treatment is a negative prognostic factor where the hazard of death increased by 2% per day of delay in the initiation of RT [16]. According to another study, the delay in starting radiation treatment might have adverse effects as they suggested a mean tumour doubling time of 24 days [17]. Hence, RT is generally started as soon as the wound heals, but our analysis did not show any effect of delay in RT beyond 28 days of survival.

In the LANDMARK randomised study, Stupp *et al* [6] reported that the delivery of temozolomide during radiotherapy increased survival, suggesting that this DNA alkylating agent can increase survival by enhancing radiosensitivity of glioblastoma cells and reported that the OS rates with radiation and temozolomide are 27.2% at 2 years, 16.0% at 3 years, 12.1% at 4 years and 9.8% at 5 years. 47% of that study population completed six cycles of chemotherapy, while in our patient population only 32.6% completed six cycles; this could be due to disease progression and death, but not due to toxicity of chemotherapy. We did not notice any grade 2 or more toxicity during the entire period which had shown that our population tolerated temozolomide well.

Both univariate and multivariate analyses retained the statistically significant improvement of OS and DFS with use of adjuvant chemotherapy.

So, our study may be favouring the trials evaluating the suggestion that adjuvant chemotherapy may be provided to all cases of glioblastoma without considering other prognostic factors.

Small sample size and inadequate data pertaining to prognostic factors like IDH and MGMT are limitations of this study. This may be due to the study being preliminary in nature in a low income country where accessibility to all resources may be limited.

## Conclusion

Among various clinical and treatment-related prognostic factors evaluated in our study, younger age at presentation and the addition of temozolomide chemotherapy to radiation showed improvement in OS and DFS. The use of the antiepileptic drug Levetiracetam had an impact on DFS in glioblastoma patients.

## Conflicts of interest

None.

## Funding

None.

## Figures and Tables

**Table 1.** Various prognostic groups and univariate analysis of factors on OS and DFS.

		*n* = 46	OS (months)	OS (months)	*p* value	DFS (months)	DFS (months)	*p* value
Prognostic factors	Groups		**mean**	**median**		**mean**	**median**	
Age	Group 1: Less than or 45 years	18	13.50	11	0.015	9.22	7.50	0.015
	Group 2: More than or 46 years	28	8.64	8		6.43	6	
Sex	Group 1: Female	20	10.75	9.50	0.993	7.20	6	0.689
	Group 2: Male	26	10.38	8		7.77	7	
Preoperative symptom presentation	Group 1: Seizures at presentation	14	13.21	11	0.191	7.50	6.50	0.443
	Group 2: Other symptoms including headache, neurological deficits and altered sensorium at presentation	32	9.38	8		7.53	6.50	
Location of tumour	Group 1: Cerebral cortex	38	10.55	8	0.952	7.11	6	0.397
	Group 2: Others like cerebellum and basal ganglia	8	10.50	10		9.50	9	
Side of lesion	Group1: Left	15	9.40	9	0.766	6.33	6	0.513
	Group 2: Right	23	11.30	8		7.61	6	
	Group 3: Midline	8	10.50	10		9.50	9	
Lobe involved	Group 1: Frontal	13	11.69	8	0.324	6.62	6	0.322
	Group 2: Parietal	12	8.08	5.5		6.42	5	
	Group 3: Temporal	13	11.69	11		8.23	9	
	Group 4: Others	8	10.50	10		9.50	9	
ECOG performance status	Group 1: 1, 2	21	12.24	10	0.110	8.57	6	0.085
	Group 2: 3, 4	25	9.12	8		6.64	7	
Anti-epileptic drug used	Group 1: Levetiracetam	18	12.07	10	0.093	8.43	7	0.027
	Group 2: Non-levetiracetam	28	8.17	7		6.11	6	
Type of surgery	Group 1: Total excision	17	11.35	8	0.607	7.94	6	0.606
	Group 2: Subtotal excision / post-operative MRI scan shows residual lesion	29	10.07	8		7.28	7	
Gap between surgery and radiation	Group 1: Surgery to radiation gap of less than or 28 days	19	11.53	8	0.868	7.63	7	0.960
	Group 2: Above or 29 days	27	9.85	8		7.44	6	
Usage of concurrent temozolomide drug	Group 1: Yes	31	11.13	11	0.237	7.74	6	0.543
	Group 2: No	15	9.33	8		7.07	7	
No. of cycles of adjuvant temozolomide	Group1: 6 cycles or more than 6 cycles	15	17.73	14	0.0001	10.67	11	0.0001
	Group 2: Less than six cycles	31	7.06	6		6	6	
